# Investigation of Extracellular Vesicles From SARS-CoV-2 Infected Specimens: A Safety Perspective

**DOI:** 10.3389/fimmu.2021.617042

**Published:** 2021-04-22

**Authors:** Yury O. Nunez Lopez, Anna Casu, Richard E. Pratley

**Affiliations:** Translational Research Institute, AdventHealth, Orlando, FL, United States

**Keywords:** extracellular vesicle, Trojan exosome, Trojan EV, SARS-CoV-2, COVID-19, laboratory safety

## Abstract

The coronavirus disease 2019 (COVID-19) pandemic, caused by the SARS-CoV-2 virus, is wreaking havoc around the world. Considering that extracellular vesicles (EVs) released from SARS-CoV-2 infected cells might play a role in a viremic phase contributing to disease progression and that standard methods for EV isolation have been reported to co-isolate viral particles, we would like to recommend the use of heightened laboratory safety measures during the isolation of EVs derived from SARS-CoV-2 infected tissue and blood from COVID-19 patients. Research needs to be conducted to better understand the role of EVs in SARS-CoV-2 infectivity, disease progression, and transmission. EV isolation procedures should include approaches for protection from SARS-CoV-2 contamination. We recommend the EV and virology scientific communities develop collaborative projects where relationships between endogenous EVs and potentially lethal enveloped viruses are addressed to better understand the risks and pathobiology involved.

## Introduction

The SARS-CoV-2 virus pandemic is wreaking havoc around the globe. A huge amount of effort and money has been directed toward studying the virus and treating the disease it causes: COVID-19. However, there remain significant gaps in our understanding of the underlying pathogenic mechanisms by which the virus causes disease. In particular, it is unknown whether extracellular vesicles (EVs) contribute to SARS-CoV-2 infection and COVID-19 progression. Indeed, a potential role of EVs in SARS-CoV-2 infection and COVID-19 progression has been recently hypothesized (1, 2).

SARS-CoV-2 infection appears to be primarily transmitted through inhalation of respiratory droplets produced by infected patients (who may or may not be symptomatic). In addition to affecting the upper respiratory tract and lungs, the virus can also be detected in the blood, intestines, heart, kidneys, liver, sperm, eyes, and possibly the brain, suggesting a viremic phase contributes to the pathophysiologic abnormalities observed in COVID-19 (3).

SARS-CoV-2 is an enveloped RNA virus that, like other enveloped viruses, uses a portion of the cell membrane to package its proteins and nucleic acids. Enveloped viruses share several characteristics with EVs, including size and physicochemical properties (4). These similarities are not accidental because, as it turns out, these viruses hijack the endocytic pathway that is normally used for EV biogenesis to assemble and secrete viral particles. EVs, in fact, have been hypothesized to be “close relatives” of enveloped viruses (5), possibly having an evolutionary relationship (e.g., viruses possibly arising as “Trojan exosomes” or by convergent evolution) (4–6).

## EXTRACELLULAR VESICLES as Carriers of Viral Particles or Viral Components (“Trojan EVs”)

Growing evidence indicates that EVs secreted by infected cells (e.g., HIV-infected and HCV-infected cells) may contain both cell-derived and virus-derived components (7–9). Viral microRNA (miRNAs), proteins, and even entire virions have been found incorporated into EVs, which could either promote or restrict viral replication in target cells (4, 10). Accumulating evidence also suggests that many non-enveloped RNA viruses undergo nonlytic release from infected cells by producing and releasing virions *via* exosome-like vesicles (11–13). These virus-laden EVs may originate from distinct types of vesicles depending on the mechanisms exploited by the infecting viruses. For example, viruses hijacking the endocytic/exosomal pathway may produce virus-loaded exosomes [e.g., exosomes containing HCV virus (14, 15)], while other viruses seem to egress *via* autophagosome-derived vesicles [e.g., EVs containing coxsackie virus (12)]. Some viruses like HIV have been shown to enhance infectivity by getting entrapped in exosome and microvesicle aggregates (16). These and additional reports underscore the importance of EVs in viral infection, which not only facilitate intercellular communication functions that can trigger host immune responses, but also play important roles in viral entry, spread, and immune evasion (17, 18).

Remarkably, EVs from HCV-infected cells contained exosomal and viral proteins and viral RNA material representing the full HCV genome and were shown to pass the infection to naive human hepatoma cells to establish a productive infection (7). This EV-mediated HCV infection was additionally shown to be partially resistant to neutralizing antibodies and independent of structural viral proteins (7). This latter result suggested that transmission through EVs contributes to the known immune evasive properties of HCV. Similarly, full virions of non-enveloped hepatitis A virus (HAV) hide inside host-derived exosome-like vesicles and become protected from antibody-mediated neutralization, which allow for efficient stealth infection and replication in the liver (19).

In another revealing series of recent experiments, O’Hara and colleagues demonstrated that human JC polyomavirus (JCPyV, a common human pathogen that, under immunosuppressed conditions, causes progressive and often fatal demyelinating multifocal leukoencephalopathy) reaches and infects brain glial cells, which do not express the viral receptors, *via* infectious JCPyV-containing EVs released by infected choroid plexus epithelial (CPE) cells (20). Notably, the viral transmission mediated by EVs was not neutralized by antisera against the virus and transmission electron microscopy of EVs purified from infected CPE cells identified distinct subpopulations of EVs, some of which had viral particles enclosed within the EVs while others had the virions attached only to the external EV surface (20). This infectious EV-mediated mechanism has been shown to be more efficient than single viral particle infection, particularly for rotaviruses and polioviruses (13, 21). On one hand, the vesicle membrane is suggested to protect/cloak its viral cargo from degradation or recognition by the immune system. On the other hand, by clustering inside vesicles, the virions are delivered to target cells at higher effective concentrations, therefore enabling high multiplicities of infection (21).

In our viewpoint, the interplay between viruses and EVs is complex, with different subpopulations of EVs probably performing antagonistic biological functions: some EV subsets from specific cell types (e.g., originating from infected cells) may enhance/spread infection and interfere with the immune system, while other EV subsets (e.g., originating from non-infected immune cells) may benefit the individual by contributing to the activation of anti-viral responses. Whether the balance of these two forces leans toward benefiting the virus or the immune system (akin a “tug-of-war” between virus-hijacked cells and immune cells) will eventually decide the health outcomes. For additional insight on how viruses manipulate EV production and secretion to increase the virus persistence, pathogenesis, and transmission, and to also gain insight into how EV response may contribute to a protective antiviral response, we recommend the excellent reviews by Wurdinger and colleagues (18), Raab-Traub and Dittmer (10), and Bello-Morales and colleagues (17).

## SARS-CoV-2 RNA Detected in the Circulation

The presence of SARS-CoV-2 RNA has been recently documented in plasma samples of a relatively large percentage of people with COVID-19 in several key clinical studies (22–25). Importantly, the levels of circulating viral RNA (RNAemia) correlated with the severity of the disease. Because available data do not support the replication of SARS-CoV-2 in peripheral blood cells, the circulating viral RNA is suggested to have originated from viral particles, viral components, or infected cells that leaked into the blood stream after the breakdown of infected lung tissue (26). However, researchers have been unable to successfully culture SARS-CoV-2 from plasma samples (27). We speculate that viral RNA components may have also originated from circulating EVs derived from infected cells loaded with specific viral cargo. In any case, “the high prevalence of RNAemia in symptomatic individuals also raises theoretical concern for occupational bloodborne exposure” (27). This issue should be considered and heightened safety measures implemented when processing blood and tissue samples from infected individuals.

## Extracellular Vesicles in COVID-19 Patients

Little evidence exists on the role of EVs on the pathogenesis of SARS-CoV-2 infection, but recent publications provide some indirect support for their potential contribution spreading infection and/or modulating the host immune response. Recently, Kwon and collaborators reported that EVs isolated from lung epithelial cells that overexpressed SARS-CoV-2 RNAs for Nsp1, Nsp12, E, and N, contained the viral RNAs and were able to transfer them to pluripotent stem cell-derived cardiomyocytes and induced the expression of proinflammatory genes IL1B, IL6, and MCP1 (28). However, this study tested supraphysiological levels of the viral genes and failed to include a negative control cell type. In addition, Zaid and colleagues reported that CD41+ platelet-derived EVs that expose anionic phospholipids such as phosphatidylserine on their surface, were increased in the blood of non-severe COVID-19 patients (but not in severe cases) and that SARS-CoV-2 RNA can associate with human platelets (29). Because, the concentration of platelet EVs could not be fully explained by the number of platelets, the authors reasoned that the reduction of platelet EVs in severe COVID-19 cases, compared to non-severe cases, might point to the consumption or sequestration of this EV subtype as the disease progresses (29). More recently, Cappellano and collaborators reported that platelet EVs, quantified by CD31 and CD41 flow cytometry on 2 independent cohorts in Italy, were significantly elevated in hospitalized patients, as compared to healthy controls. Importantly, a proteomics profiling study of large EVs isolated from 53 hospitalized COVID-19 patients in China, found that alterations in pro-inflammatory, coagulation, and endothelial injury protein cargo in the large EVs may contribute to severe COVID-19 (30). On the other hand, Yu and collaborators reported that pseudotyped SARS-CoV-2 and HIV-based lentivirus induces the production of EVs with adaptive immunity-like antiviral functions in murine hypothalamic neural stem cells (31). Although the evidence is minimal and far from being comprehensive and convincing, the potential involvement of EVs in the pathogenesis of SARS-CoV-2 infection warrants further investigation.

## EV-Mediated Transfer of ACE2 and Potential Hijacking of the Endocytic/Secretory Pathway by SARS Coronaviruses

Recent work by Wang and colleagues demonstrated that EVs released by endothelial progenitors cells that overexpressed angiotensin converting enzyme 2 (ACE2, the receptor for SARS-CoV and SARS-CoV-2 coronaviruses) can transfer the receptor to recipient cells and impact biological functions (32). As suggested by other authors, transfer of ACE2-containing EVs may render recipient cells susceptible to virus docking and contribute to SARS-CoV-2 internalization and infection (1, 2).

In a recent editorial published in the European Respiratory Journal (33), Dr. Masson elaborated on the pathogenesis of COVID-19 from a cell biology perspective. In this perspective, the author highlighted electron micrographs from previous work with SARS-CoV that portrays viral particles inside double membrane vesicles inside human alveolar type II cells infected with the virus. Similarly, using electron microscopy to study ultrathin sections of SARS-CoV-2 infected human airway epithelium, Zhu and colleagues demonstrated cytoplasmic membrane-bound vesicles containing virus-like particles (34). Electron micrographs contributed by Menter and collaborators for two COVID-19 cases with a short (<12 hours) postmortem period also detected multiple vesicles containing several small virus-like particles in the cytoplasm of a podocyte, an activated glomerular endothelial cell, and a proximal tubular epithelial cell (35).

In our opinion, these membrane-bound multivesicular structures containing virus-like particles are reminiscent of multivesicular bodies (MVBs) involved in exosome biogenesis. MVBs are late endosomes that produce smaller intraluminal vesicles (ILVs) by inward budding of their limiting membrane (36). The ILVs eventually become exosomes when they are secreted into the extracellular space by fusion of the MBVs with the plasma membrane (36). Some enveloped viruses are known to hijack the endocytic pathway to replicate and escape in a non-lytic fashion *via* an “Endosomal Sorting Complexes Required for Transport” (ESCRT)-mediated, exosome-like mechanism involving the budding into MVBs (19, 37–39).

**Figure 1:**
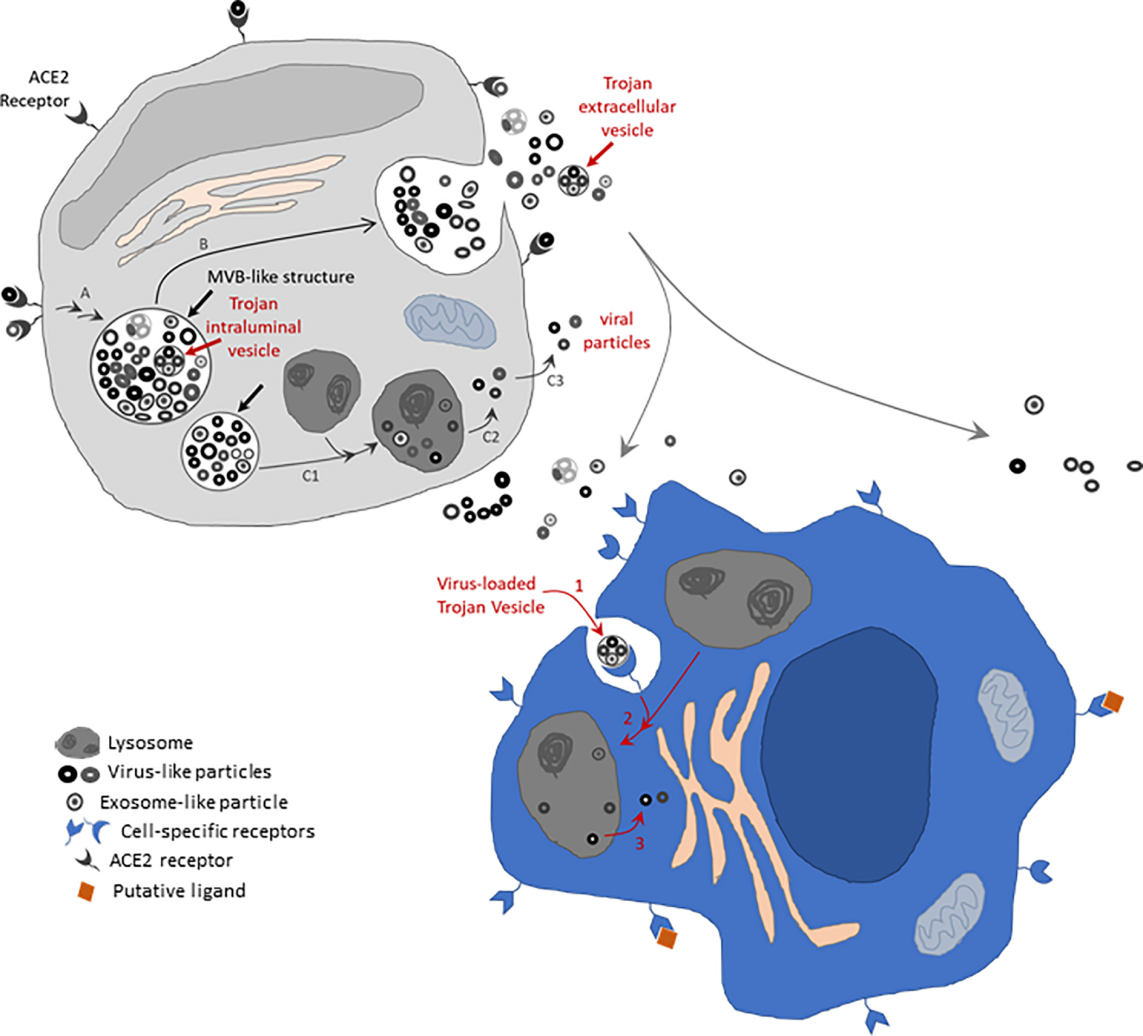
Schematic of EV-mediated mechanism potentially contributing to SARS-CoV-2 infectivity. Membrane bound structures containing virus-like and exosome-like particles of distinct electron densities and diameters (thick straight black arrows) are shown in the putative grey cell, resembling those structures identified by electron microscopy by Zhu and colleagues (34). These structures resemble multivesicular body (MVB)-like structures. The thick straight red arrow indicates an intraluminal vesicle-like structure that contains smaller nanoparticles [reminiscent of intraluminal vesicles possibly containing hidden viral particles –this also resembles a structure apparently observable in electron micrograph from (34)]. These MVB-like structures might have originated *via* the endosomal pathway after initial internalization of SARS-CoV-2 particles *via* the ACE2 receptor in expressing cells (entry route A). The MVB-like structures might proceed to fuse with the cell membrane, therefore spilling their content in the extracellular space (route B). Alternatively, they might fuse with lysosomes and consequently allow scape of the viral particles as suggested by (40) (route C1-C2-C3). Released Trojan extracellular vesicles (EVs) carrying virus-like particles may potentially be internalized by cells not expressing the ACE2 receptor but *via* cell-specific receptors that recognize specific membrane markers in the Trojan EVs. Internalized Trojan EVs may potentially infect the invaded cells (putative blue cell) by exploiting a mouse hepatitis virus (MHV)-like mechanism (40) *via* fusion with lysosomes (route 1-2-3 in blue cell). Drawings not done to scale. Membrane proteins expected to be present in viral particles and EVs that are expected to contribute to viral and Trojan EV docking are not drawn. Receptors are drawn as exaggeratedly large features on the cell surface to highlight known (ACE2 receptor-mediated) and potential (Trojan EV receptor-mediated) receptor-specific mechanisms. Trojan EVs might further contribute to SARS-CoV-2 infectivity by transferring ACE2 receptors and other viral molecules to non-expressing cells (not drawn).

Interestingly, the electron micrograph presented in Figure 3B of reference (34) (https://www.nejm.org/na101/home/literatum/publisher/mms/journals/content/nejm/2020/nejm_2020.382.issue-8/nejmoa2001017/20200214/images/img_medium/nejmoa2001017_f3.jpeg), suggests the presence of nanovesicles and/or viral particles with different electron densities, sizes, and general morphology inside the multivesicular structures (black arrows). Inside one of those multivesicular structures, we can observe what appears to be at least one larger intraluminal vesicle-like structure that contain smaller nanoparticles (see schematic in **Figure 1**). This may indicate the colocalization of viral particles and exosomes in such multivesicular body-like structures, which raises the possibility that SARS-CoV-2 may also exploit a Trojan EV strategy to replicate and spread viral infection and/or alter the cargo of bonafide exosomes to change the dynamics of the long distance crosstalk among tissues and organs in the infected individuals. High resolution transmission electron micrographs presented by Dittmayer and colleagues in Figure C and D (41) (https://pubmed.ncbi.nlm.nih.gov/33031763/#&gid=article-figures&pid=figure-uid-0) displaying one or two SARS-CoV-2 viral particles inside a relatively small vesicle of approximately 200 – 250 nm in diameter might also represent potential Trojan vesicles, provided that they reach the extracellular space as a complete unit. Confirmation of the existence of such SARS-CoV-2-laden Trojan vesicles in the extracellular space and/or the patient’s circulation, using complementary and more robust techniques such as immuno-gold electron microscopy, is necessary and important to define whether this alternative route of viral spread is possible in COVID-19 patients.

While this perspective was submitted for publication, the Altan-Bonnet group published novel findings describing an alternative lysosome-mediated exit route for β-coronaviruses (40). Using mouse hepatitis virus (MHV), which could be studied in a biosafety level 2 (BSL2) environment, the authors demonstrated that MHV associated with lysosomes and/or atypical late endosomes in infected cells. In their *in vitro* experiments, SARS-CoV-2 seemed to follow a similar route in infected Vero-E6 cells. Although their multiple experimental approaches strongly suggest a main lysosomal route of MHV egress *in vitro*, this study does not rule out the use of alternative endosomal secretion mechanisms for SARS-CoV-2 *in vivo*. We base our reasoning, in part, on the lack of observation, in published literature presenting electron microscopy images of SARS-CoV-2 infected human tissues (34, 35, 41–43), of the characteristic ultrastructural features described by Ghosh and collaborators (40), which identified viral-like particles inside lysosome-like organelles in infected cells *in vitro*. In addition, definite proof showing colocalization of SARS-CoV-2 protein markers in lysosome-like organelles using immunoelectron microscopy is lacking. Therefore, we consider the possibility that both lysosomal mediated and endosomal mediated mechanisms might play independent parallel roles in SARS-CoV-2 infection, with one mechanism or the other being potentially favored under specific tissue microenvironmental conditions. The mutational/genetic landscape of the virus could also, in principle, contribute to a varied cellular and pathophysiological host response to SARS-CoV-2 infection *in vivo*.

## Co-Isolation of Viral Particles During EV Purification

Because of their shared intracellular biogenesis mechanisms, viruses (enveloped viruses in particular) and EVs have important similarities, including comparable size and buoyant density, that make their complete experimental separation very difficult (5), at least when using common techniques applied in the EV field such as density gradients and ultracentrifugation, size exclusion chromatography, and polymer precipitation-based methods.

As an example, Chugh and colleagues reported that Kaposi’s sarcoma-associated herpesvirus (KSHV) virions copurified with EVs while using centrifugation-based protocols (i.e., differential ultracentrifugation, sucrose gradients, and ExoQuick polymer precipitation) designed to enrich for circulating exosomal fractions derived from KSHV-associated malignancies (44). These authors were only able to eliminate the viral particles when positive selection affinity beads were used to pull down EVs based on an EV-specific surface marker (i.e., CD63) not present in the virion’s surface. Because KSHV virions are relatively large, with 180 nm diameter, and tend to aggregate, filtration of the EV preparations through a 0.2 μm filter also eliminated most of the KSHV load in this case. However, even when the viral particles had been removed, the EV preparations contained functional KSHV-encoded miRNAs that were able to enhance cell migration and IL6 secretion in challenged hTERT-HUVEC (44). This latter result additionally supports the idea that EVs secreted by infected cells could be functional and contribute to the pathogenesis of the viral infection.

**Figure 2:**
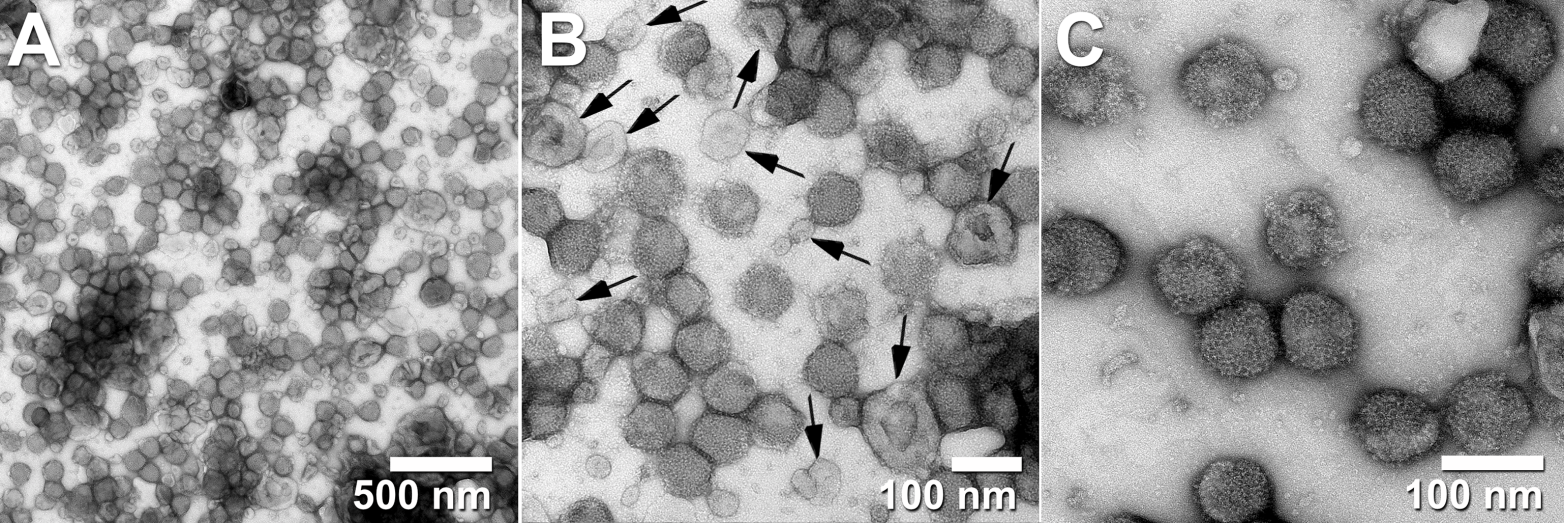
Transmission electron micrographs of influenza virus A/Puerto Rico/8/34 H1N1 purified with steric exclusion chromatography (SXC) and pseudo-affinity chromatography with a sulfated cellulose membrane adsorber. Co-eluted exosome-like vesicles are visible in panels **(A, B)** and labeled with black arrows in panel **(B)**. Panel **(C)** displays homogeneous (mostly intact) viral particles. Black arrows were added to the original published figure to highlight examples of extracellular vesicles (with lighter electron-density and collapsed cup shape) side by side with intact viral particles (non-collapsed round particles with higher electron density). All images are from the same sample at different magnifications. Pictures taken by Dietmar Riedel from the Max-Planck-Institute for Biophysical Chemistry in Göttingen, Germany. Reproduced with permission from Dr.-Ing. Pavel A Marichal-Gallardo, Max-Planck-Institute for Biophysical Chemistry in Göttingen, Germany (license CC BY-NC-SA 4.0).

In work developing steric exclusion chromatography (SXC) as a new platform for the purification of large biomolecules such as virus particles (e.g., influenza, yellow fever, and adeno-associated viruses), Marichal-Gallardo and collaborators were able to produce full yield of infectious titer with residual DNA and protein contamination levels that were below regulatory requirements for vaccine development (45). Yet, in excellent transmission electron micrographs (**Figure 2**), they show that EVs from the host cells were often co-purified with the target virus particles (45). This again, confirms the difficulties of separating EVs from viral particles using a variety of methods.

As Drs. Nolte-’t Hoen, Cremer, Gallo, and Margolis wrote on their PNAS perspective back in 2016: “Unless more specifically defined, it is currently virtually impossible to specifically separate and identify EVs that carry viral proteins, host proteins, and viral genomic elements from enveloped viral particles that carry the same molecules.” (5) In our opinion, this statement remains accurate today. This raises our concerns for the likely co-purification of SARS-CoV-2 viral particles in EV preparations produced from infected samples.

## Final Remarks and Recommendations

We reason that current methods for EV isolation would likely co-isolate SARS-CoV-2 particles and therefore applying these procedures on COVID-19 samples may require heightened laboratory safety standards. In addition, the possibility of alternative modes of viral particle or viral component transmission *via* Trojan EVs compounds the associated risks. However, to our knowledge, no recommendations for increased lab safety standards while isolating EVs from COVID-19 (or any other lethal viral disease) samples have been published or disseminated as of yet.

The publication from the U.S. Department of Health and Human Services: Biosafety in Microbiological and Biomedical Laboratories, 5^th^ Edition, recommends that, while working with samples infected with SARS-CoV, the coronavirus agent causative of the 2002-2003 SARS pandemic, activities involving the manipulation of untreated specimens should be performed in BSL2 facilities following BSL3 practices and that any procedure with the potential to generate aerosols should be performed in a biosafety cabinet (46). It is also stated that SARS-CoV propagation in cell culture and the initial characterization of viral agents recovered in cultures of SARS specimens must be performed in a BSL3 facility using BSL3 practices and procedures, with risk assessment dictating the additional use of respiratory protection (46). Regarding the more recent SARS-CoV-2 pandemic agent, interim guidelines from the CDC (47) and WHO (48) recommend that procedures such as virus isolation are to be performed in a BSL3 environment. Although the isolation of exosomes and EVs in general is not specifically mentioned in these documents, it is our opinion that important risks may exist, considering that viruses and other infectious particles could be co-isolated with exosomes and EVs.

We strongly recommend the implementation of approaches to protect against contamination with viral particles during the isolation of EVs derived from tissues and blood products infected with SARS-CoV-2 or other lethal viruses, in general. Primary hazards important to consider when working with any high biosafety level agents include autoinoculation, exposure to aerosols, and ingestion. Those risks could be minimized by using physical containment devices such as biosafety cabinets of appropriate containment level. Biosafety cabinets should be used, whenever possible, for any aerosol-generating procedure and additional containment devices used during procedures with high potential to create aerosols (e.g., centrifugation, homogenization). Because of the potential for aerosol formation during EV purification, we specifically recommend that this type of work be conducted in, at least, a BSL2 environment with unidirectional airflow and manipulations performed using BSL3-like precautions, somewhat emulating standards often applied to a variety of virus isolation protocols. Centrifugation steps, should be conducted using centrifuge safety buckets or sealed centrifuge tubes in sealed rotors. Tube opening during EV purification and manipulation of samples during EV characterization procedures that measure intact EV particles (e.g., nanoparticle tracking analysis using the NanoSight, where sample loading using standard 1 mL syringes is common practice and sample droplets may be produced and released into the environment during the removal of air bubbles inside the syringe) should be carefully conducted inside a -Class II biosafety cabinet with proper decontamination of the cabinet and equipment surfaces afterward. For downstream experiments that do not require intact EVs (e.g., proteomics, metabolomics, and RNA profiling), EV preparations should be lysed or accordingly inactivated immediately after small aliquots of intact EVs (if needed) are properly stored. As Jureka and collaborators recently demonstrated, SARS-CoV-2 particles can be effectively inactivated by TRIzol, 10% neutral buffered formalin (at concentrations ranging from 0.5% to 2% total formaldehyde concentration after 1 h at room temperature), beta-propiolactone (incubation with 0.5% beta-propiolactone for 16 h at 4 °C followed by 2 h incubation at 37 °C), and heat (5 min at 100°C and 45 min at 56°C). These, in fact, represent key reagents to safely conduct downstream characterization (e.g., RNA isolation for gene expression, microscopy studies, immunological assays/vaccine preparation, and Western blotting respectively) of SARS-CoV-2 EV preparations. Lysed or inactivated EVs should not pose health risks needing more than standard laboratory safety measures. However, cell culture experiments using intact EV preparations purified from SARS-CoV-2 infected samples should be considered with extra care (because of the potential for propagation of intact co-isolated viral particles and/or Trojan EVs) and be conducted in a BSL3 environment. By the same token, EV research using samples infected with other lethal respiratory viruses, should also be conducted under heightened laboratory safety standards following appropriate biosafety training and requiring appropriate containment devices and facilities. For methods and precautions used in a BSL3 environment, we recommend the Biological Safety BSL3 Laboratory Manual (https://ehs.yale.edu/sites/default/files/files/bsl3-lab-manual.pdf). Because of the potential risks described here, it is advisable that any researcher performing purification/isolation of EVs from SARS-CoV-2 (or other lethal viruses) infected samples demonstrates knowledge of basic and advanced biosafety laboratory skills. Guidance from the institutional biosafety officers should be sought to define appropriate containment methods, safety procedures, and protocols applicable for the specific projects. A summary of our main recommendations is presented in **Table 1**.

**Table 1:** Biosafety recommendations for work involving the isolation and use of extracellular vesicles (EVs) from SARS-CoV-2 infected samples.

Training	Advanced biosafety training must be provided to laboratory personnel conducting research with SARS-CoV-2 infectious material. Seek support from the institutional office of environmental health and safety (EHS).
Personal Protective Equipment (PPE)	Recommended use of rear-opening lab coat, double gloves, face mask, and goggles. Face shield may also be advisable. Reusable PPE should be decontaminated or autoclaved (e.g., clothes or linens prior to laundering).
Aerosol Containment	Manipulation of SARS-CoV-2 infected materials should be conducted inside a Class II (or better) biological safety cabinet (BSC). Avoid the generation of aerosols outside the BSC.
Centrifugation	Should be conducted using centrifuge safety buckets or sealed centrifuge tubes in sealed rotors.
Nanoparticle Tracking Analysis	To avoid the generation of aerosols with contaminating live viral particles (e.g., during elimination of air bubbles from the loading syringe before running samples through the NanoSight), it is recommended to use inactivated EVs to measure the size and concentration of the particles.
Decontamination	Contaminated laboratory waste and equipment should be decontaminated, e.g., autoclaved or decontaminated with fresh solution of 10% chlorine bleach (final concentration against the volume of waste) before disposal or washing. Liquid waste decontaminated with 10% chlorine bleach should be allowed at least 30 minutes of contact time. If using 10% bleach solution on work surfaces and equipment, allow it to air dry then wipe with 70% ethanol to prevent rusting of stainless steel surfaces.
Virus inactivation (49)	To minimize exposure risk, virus inactivation in samples/aliquots not needed for live EV experiments is recommended as soon as preparations are generated: For nucleic acid assays: lyse EV preparations in TRIzol (at least at a 10% final concentration and with 10 minutes incubation at room temperature), and freeze at -80 °C until use. For immunohistochemistry and microscopy: fixed samples in paraformaldehyde (e.g., at least at 1% final formaldehyde concentration with 1 hour or longer incubation at room temperature). For biochemical assay development: incubate with 0.5% beta-propiolactone for 16 hours at 4 °C followed by 2 hours incubation at 37 °C), or apply heat for 5 minutes at 100 °C or 45 minutes at 56 °C).
Laboratory accidents	Should be immediately reported to the Principal Investigator and EHS. Maintain a biological spill kit in the laboratory to facilitate spill response and decontamination.
Airflow	In addition to the use of biosafety cabinet, inward directional room airflow is advisable.
Immunizations	Vaccines should be obtained for those engaged in research with potential exposure to the specific infectious agent.

For a comprehensive review of advanced biosafety laboratory procedures, we recommend the Biological Safety BSL3 Laboratory Manual from Yale University (https://ehs.yale.edu/sites/default/files/files/bsl3-lab-manual.pdf).

The publication of original studies addressing the role of EVs in SARS-CoV-2 infectivity and COVID-19 progression has been minimal until now. However, we expect that research efforts will grow quickly as has been the case with other COVID-19 research. The safety of all our colleagues involved or to be involved in the coming future in EV research with COVID-19 and other diseases caused by lethal respiratory viruses is an important issue to which we want to call attention.

## Data Availability Statement

The original contributions presented in the study are included in the article/supplementary material. Further inquiries can be directed to the corresponding author.

## Author Contributions

YN, AC, and RP equally contributed to designing, writing, and reviewing this manuscript. All authors contributed to the article and approved the submitted version.

## Conflict of Interest

The authors declare that the research was conducted in the absence of any commercial or financial relationships that could be construed as a potential conflict of interest.
